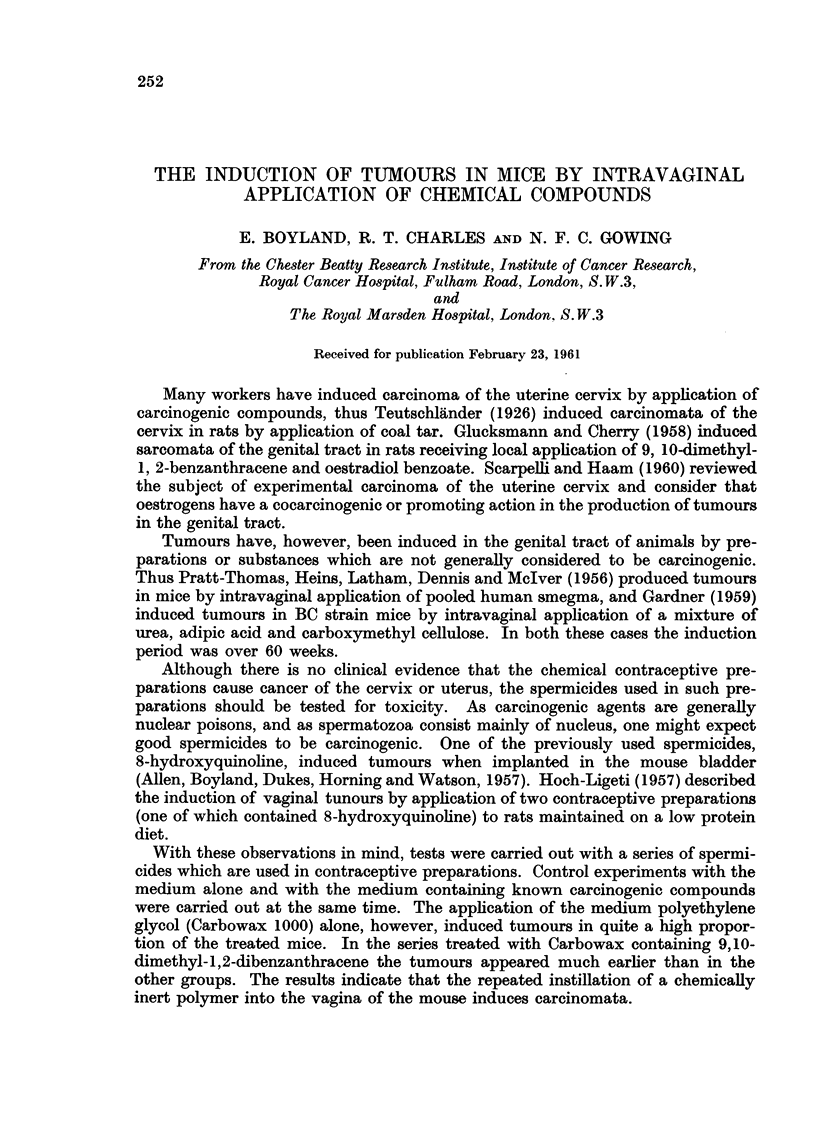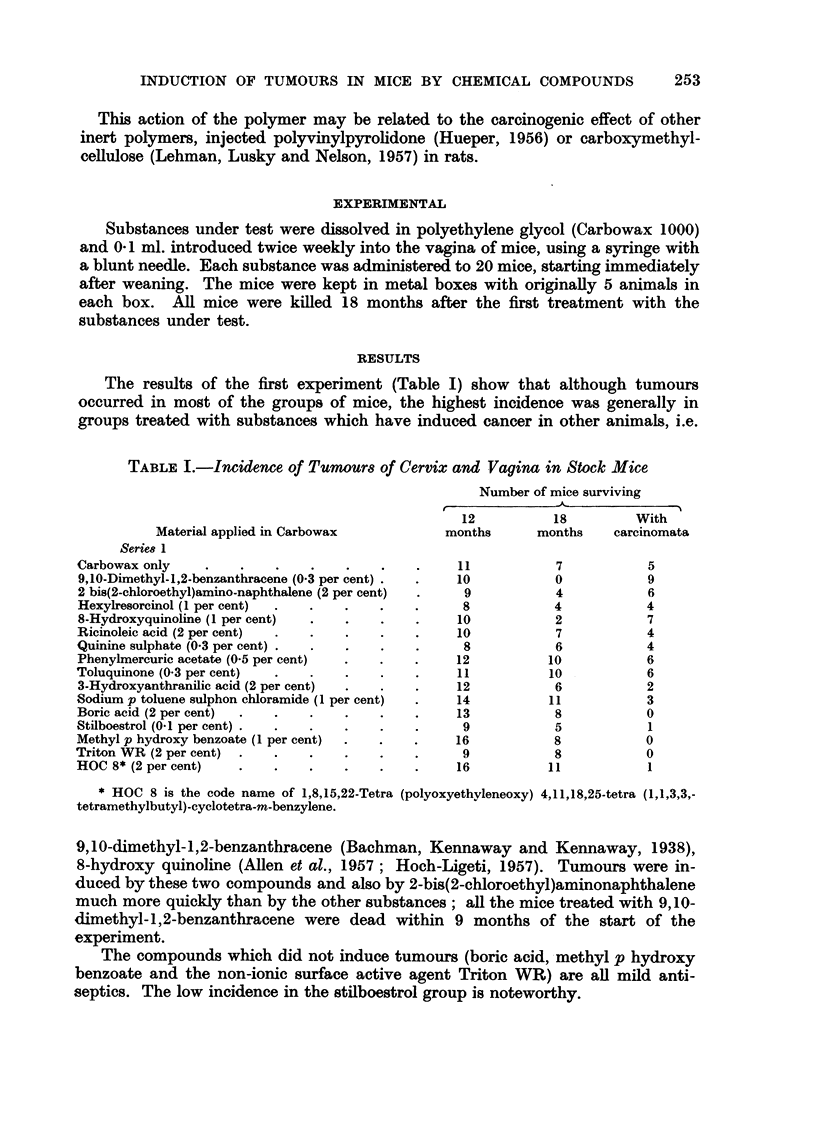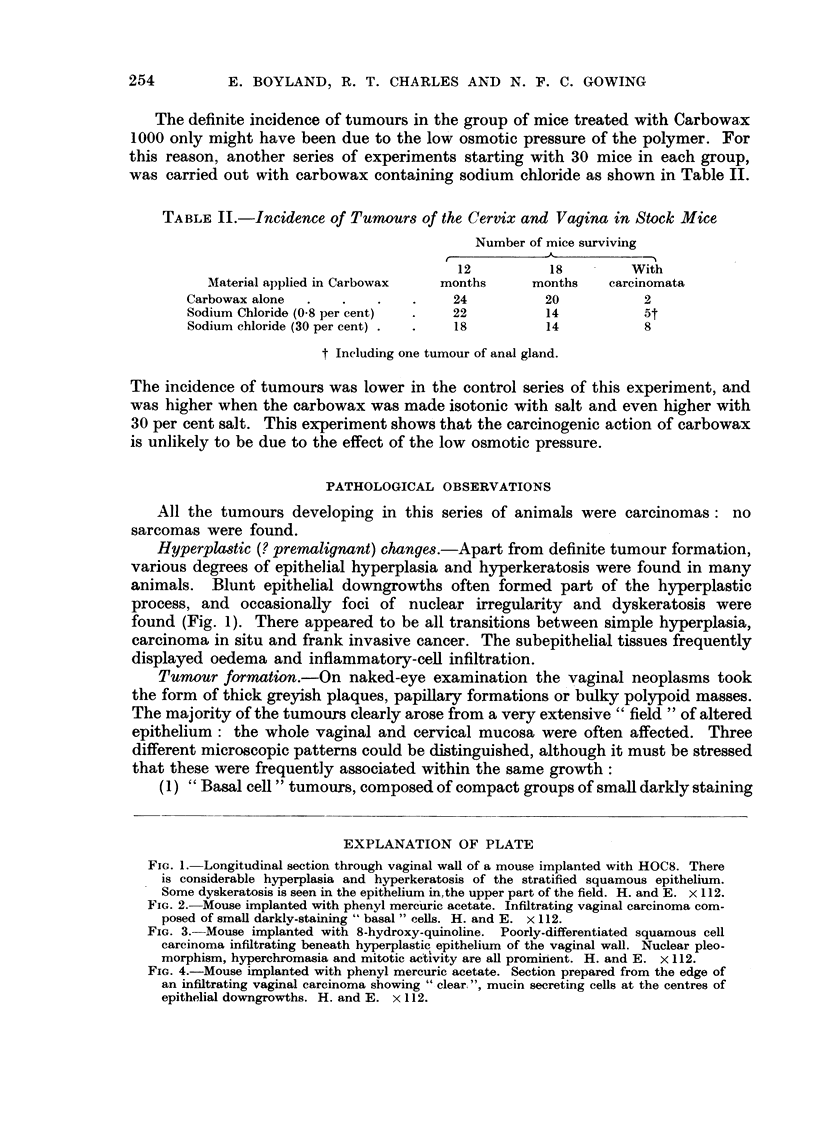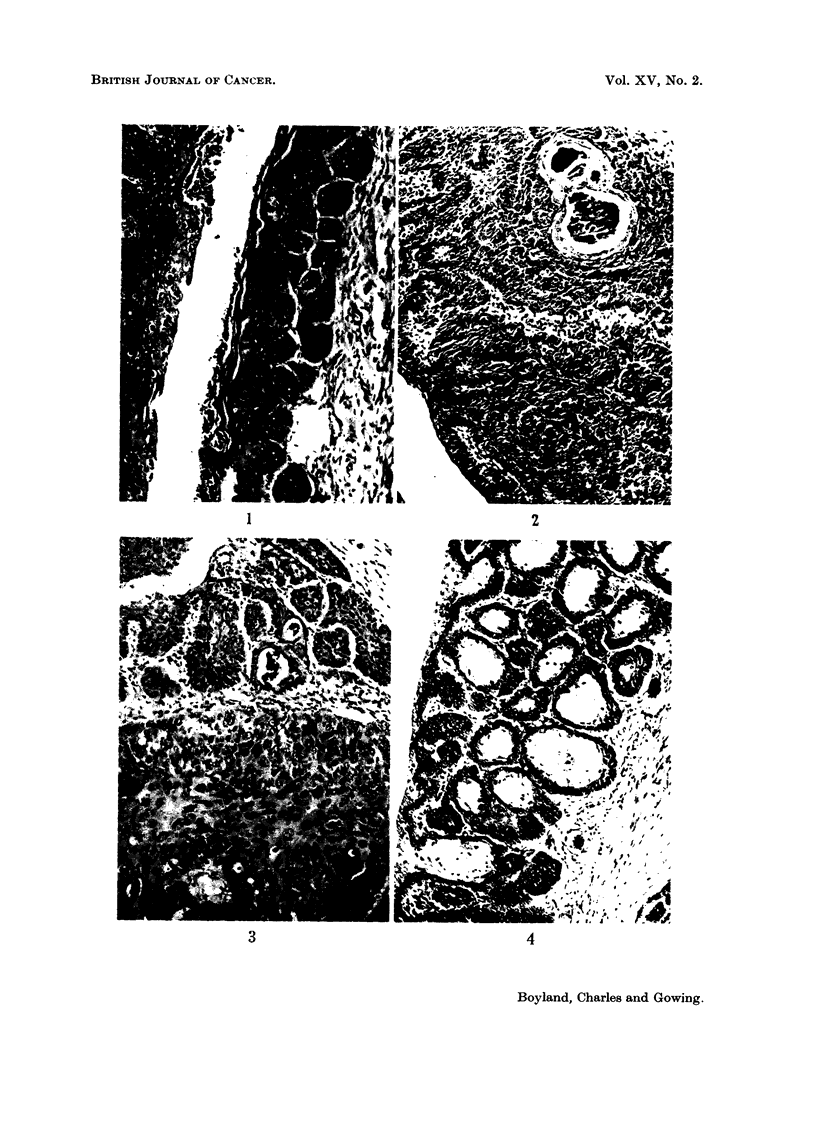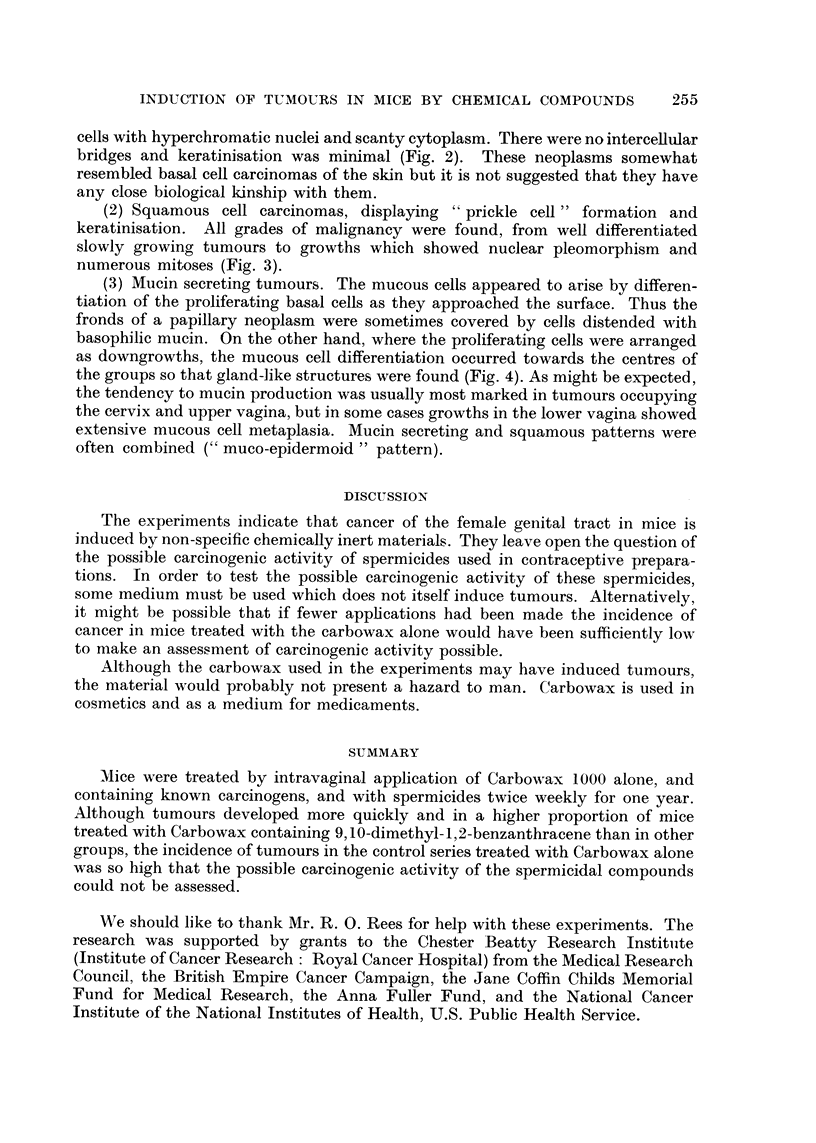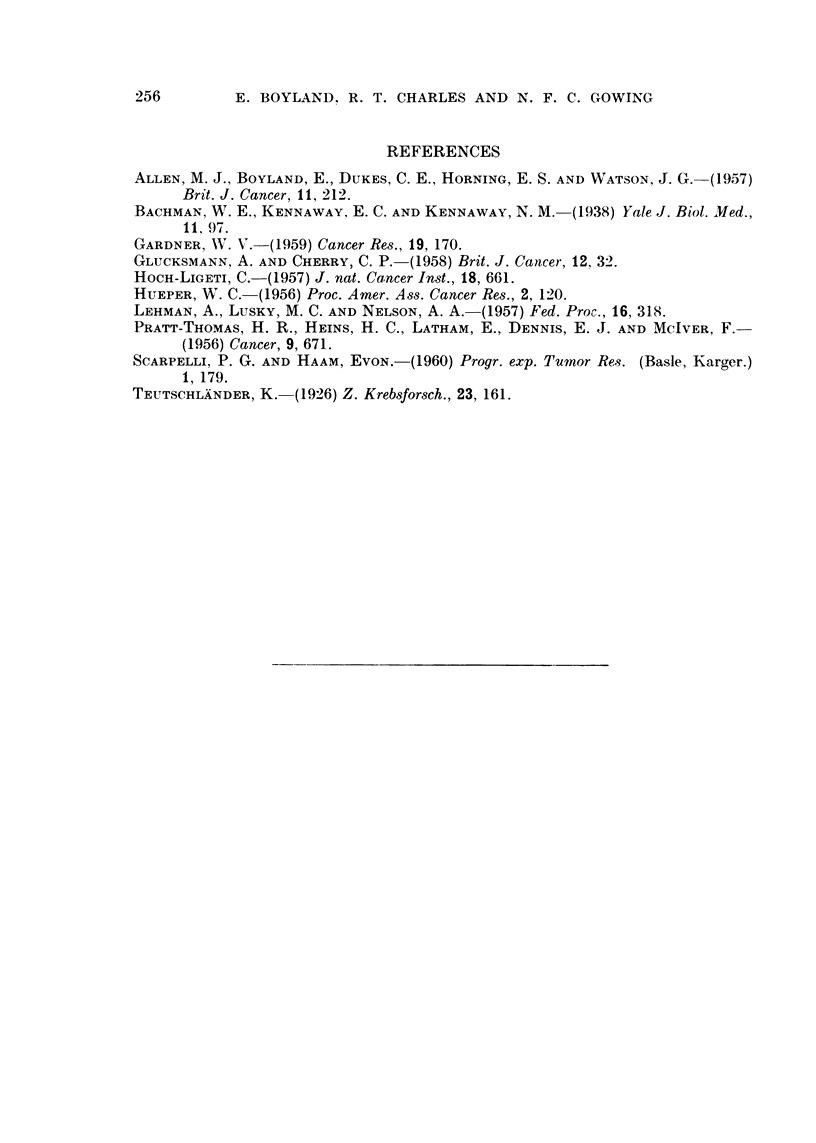# The Induction of Tumours in Mice by Intravaginal Application of Chemical Compounds

**DOI:** 10.1038/bjc.1961.31

**Published:** 1961-06

**Authors:** E. Boyland, R. T. Charles, N. F. C. Gowing

## Abstract

**Images:**


### THE INDUCTION OF TUMOURS IN MICE BY INTRAVAGINAL APPLICATION OF CHEMICAL COMPOUNDS

E. BOYLAND, R. T. CHARLES AND N. F. C. GOWING

*From the Chester Beatty Research Institute, Institute of Cancer Research, Royal Cancer Hospital, Fulham Road, London, S.W.3,*
*and*
*The Royal Marsden Hospital, London, S.W.3*

Received for publication February 23, 1961

Many workers have induced carcinoma of the uterine cervix by application of carcinogenic compounds, thus Teutschländer (1926) induced carcinomata of the cervix in rats by application of coal tar. Glucksmann and Cherry (1958) induced sarcomata of the genital tract in rats receiving local application of 9, 10-dimethyl-1, 2-benzanthracene and oestradiol benzoate. Scarpelli and Haam (1960) reviewed the subject of experimental carcinoma of the uterine cervix and consider that oestrogens have a cocarcinogenic or promoting action in the production of tumours in the genital tract.

Tumours have, however, been induced in the genital tract of animals by preparations or substances which are not generally considered to be carcinogenic. Thus Pratt-Thomas, Heins, Latham, Dennis and McIver (1956) produced tumours in mice by intravaginal application of pooled human smegma, and Gardner (1959) induced tumours in BC strain mice by intravaginal application of a mixture of urea, adipic acid and carboxymethyl cellulose. In both these cases the induction period was over 60 weeks.

Although there is no clinical evidence that the chemical contraceptive preparations cause cancer of the cervix or uterus, the spermicides used in such preparations should be tested for toxicity. As carcinogenic agents are generally nuclear poisons, and as spermatozoa consist mainly of nucleus, one might expect good spermicides to be carcinogenic. One of the previously used spermicides, 8-hydroxyquinoline, induced tumours when implanted in the mouse bladder (Allen, Boyland, Dukes, Horning and Watson, 1957). Hoch-Ligeti (1957) described the induction of vaginal tunours by application of two contraceptive preparations (one of which contained 8-hydroxyquinoline) to rats maintained on a low protein diet.

With these observations in mind, tests were carried out with a series of spermicides which are used in contraceptive preparations. Control experiments with the medium alone and with the medium containing known carcinogenic compounds were carried out at the same time. The application of the medium polyethylene glycol (Carbowax 1000) alone, however, induced tumours in quite a high proportion of the treated mice. In the series treated with Carbowax containing 9,10-dimethyl-1,2-dibenzanthracene the tumours appeared much earlier than in the other groups. The results indicate that the repeated instillation of a chemically inert polymer into the vagina of the mouse induces carcinomata.

This action of the polymer may be related to the carcinogenic effect of other inert polymers, injected polyvinylpyrolidone (Hueper, 1956) or carboxymethylcellulose (Lehman, Lusky and Nelson, 1957) in rats.

### EXPERIMENTAL

Substances under test were dissolved in polyethylene glycol (Carbowax 1000) and 0·1 ml. introduced twice weekly into the vagina of mice, using a syringe with a blunt needle. Each substance was administered to 20 mice, starting immediately after weaning. The mice were kept in metal boxes with originally 5 animals in each box. All mice were killed 18 months after the first treatment with the substances under test.

### RESULTS

The results of the first experiment (Table I) show that although tumours occurred in most of the groups of mice, the highest incidence was generally in groups treated with substances which have induced cancer in other animals, i.e.

TABLE I.—*Incidence of Tumours of Cervix and Vagina in Stock Mice*

| Material applied in Carbowax | Number of mice surviving | | |
|---|---|---|---|
| | 12 months | 18 months | With carcinomata |
| *Series* 1 | | | |
| Carbowax only | 11 | 7 | 5 |
| 9,10-Dimethyl-1,2-benzanthracene (0·3 per cent) | 10 | 0 | 9 |
| 2 bis(2-chloroethyl)amino-naphthalene (2 per cent) | 9 | 4 | 6 |
| Hexylresorcinol (1 per cent) | 8 | 4 | 4 |
| 8-Hydroxyquinoline (1 per cent) | 10 | 2 | 7 |
| Ricinoleic acid (2 per cent) | 10 | 7 | 4 |
| Quinine sulphate (0·3 per cent) | 8 | 6 | 4 |
| Phenylmercuric acetate (0·5 per cent) | 12 | 10 | 6 |
| Toluquinone (0·3 per cent) | 11 | 10 | 6 |
| 3-Hydroxyanthranilic acid (2 per cent) | 12 | 6 | 2 |
| Sodium *p* toluene sulphon chloramide (1 per cent) | 14 | 11 | 3 |
| Boric acid (2 per cent) | 13 | 8 | 0 |
| Stilboestrol (0·1 per cent) | 9 | 5 | 1 |
| Methyl *p* hydroxy benzoate (1 per cent) | 16 | 8 | 0 |
| Triton WR (2 per cent) | 9 | 8 | 0 |
| HOC 8* (2 per cent) | 16 | 11 | 1 |

* HOC 8 is the code name of 1,8,15,22-Tetra (polyoxyethyleneoxy) 4,11,18,25-tetra (1,1,3,3,-tetramethylbutyl)-cyclotetra-*m*-benzylene.

9,10-dimethyl-1,2-benzanthracene (Bachman, Kennaway and Kennaway, 1938), 8-hydroxy quinoline (Allen *et al.*, 1957 ; Hoch-Ligeti, 1957). Tumours were induced by these two compounds and also by 2-bis(2-chloroethyl)aminonaphthalene much more quickly than by the other substances ; all the mice treated with 9,10-dimethyl-1,2-benzanthracene were dead within 9 months of the start of the experiment.

The compounds which did not induce tumours (boric acid, methyl *p* hydroxy benzoate and the non-ionic surface active agent Triton WR) are all mild antiseptics. The low incidence in the stilboestrol group is noteworthy.

The definite incidence of tumours in the group of mice treated with Carbowax 1000 only might have been due to the low osmotic pressure of the polymer. For this reason, another series of experiments starting with 30 mice in each group, was carried out with carbowax containing sodium chloride as shown in Table II.

TABLE II.—*Incidence of Tumours of the Cervix and Vagina in Stock Mice*

| Material applied in Carbowax | Number of mice surviving: 12 months | 18 months | With carcinomata |
|---|---|---|---|
| Carbowax alone . . . . | 24 | 20 | 2 |
| Sodium Chloride (0·8 per cent) . | 22 | 14 | 5† |
| Sodium chloride (30 per cent) . . | 18 | 14 | 8 |

† Including one tumour of anal gland.

The incidence of tumours was lower in the control series of this experiment, and was higher when the carbowax was made isotonic with salt and even higher with 30 per cent salt. This experiment shows that the carcinogenic action of carbowax is unlikely to be due to the effect of the low osmotic pressure.

### PATHOLOGICAL OBSERVATIONS

All the tumours developing in this series of animals were carcinomas : no sarcomas were found.

*Hyperplastic (? premalignant) changes.*—Apart from definite tumour formation, various degrees of epithelial hyperplasia and hyperkeratosis were found in many animals. Blunt epithelial downgrowths often formed part of the hyperplastic process, and occasionally foci of nuclear irregularity and dyskeratosis were found (Fig. 1). There appeared to be all transitions between simple hyperplasia, carcinoma in situ and frank invasive cancer. The subepithelial tissues frequently displayed oedema and inflammatory-cell infiltration.

*Tumour formation.*—On naked-eye examination the vaginal neoplasms took the form of thick greyish plaques, papillary formations or bulky polypoid masses. The majority of the tumours clearly arose from a very extensive " field " of altered epithelium : the whole vaginal and cervical mucosa were often affected. Three different microscopic patterns could be distinguished, although it must be stressed that these were frequently associated within the same growth :

(1) " Basal cell " tumours, composed of compact groups of small darkly staining

---

### EXPLANATION OF PLATE

FIG. 1.—Longitudinal section through vaginal wall of a mouse implanted with HOC8. There is considerable hyperplasia and hyperkeratosis of the stratified squamous epithelium. Some dyskeratosis is seen in the epithelium in the upper part of the field. H. and E. ×112.

FIG. 2.—Mouse implanted with phenyl mercuric acetate. Infiltrating vaginal carcinoma composed of small darkly-staining " basal " cells. H. and E. ×112.

FIG. 3.—Mouse implanted with 8-hydroxy-quinoline. Poorly-differentiated squamous cell carcinoma infiltrating beneath hyperplastic epithelium of the vaginal wall. Nuclear pleomorphism, hyperchromasia and mitotic activity are all prominent. H. and E. ×112.

FIG. 4.—Mouse implanted with phenyl mercuric acetate. Section prepared from the edge of an infiltrating vaginal carcinoma showing " clear ", mucin secreting cells at the centres of epithelial downgrowths. H. and E. ×112.

1

2

3

4

Boyland, Charles and Gowing.

cells with hyperchromatic nuclei and scanty cytoplasm. There were no intercellular bridges and keratinisation was minimal (Fig. 2). These neoplasms somewhat resembled basal cell carcinomas of the skin but it is not suggested that they have any close biological kinship with them.

(2) Squamous cell carcinomas, displaying " prickle cell " formation and keratinisation. All grades of malignancy were found, from well differentiated slowly growing tumours to growths which showed nuclear pleomorphism and numerous mitoses (Fig. 3).

(3) Mucin secreting tumours. The mucous cells appeared to arise by differentiation of the proliferating basal cells as they approached the surface. Thus the fronds of a papillary neoplasm were sometimes covered by cells distended with basophilic mucin. On the other hand, where the proliferating cells were arranged as downgrowths, the mucous cell differentiation occurred towards the centres of the groups so that gland-like structures were found (Fig. 4). As might be expected, the tendency to mucin production was usually most marked in tumours occupying the cervix and upper vagina, but in some cases growths in the lower vagina showed extensive mucous cell metaplasia. Mucin secreting and squamous patterns were often combined (" muco-epidermoid " pattern).

### DISCUSSION

The experiments indicate that cancer of the female genital tract in mice is induced by non-specific chemically inert materials. They leave open the question of the possible carcinogenic activity of spermicides used in contraceptive preparations. In order to test the possible carcinogenic activity of these spermicides, some medium must be used which does not itself induce tumours. Alternatively, it might be possible that if fewer applications had been made the incidence of cancer in mice treated with the carbowax alone would have been sufficiently low to make an assessment of carcinogenic activity possible.

Although the carbowax used in the experiments may have induced tumours, the material would probably not present a hazard to man. Carbowax is used in cosmetics and as a medium for medicaments.

### SUMMARY

Mice were treated by intravaginal application of Carbowax 1000 alone, and containing known carcinogens, and with spermicides twice weekly for one year. Although tumours developed more quickly and in a higher proportion of mice treated with Carbowax containing 9,10-dimethyl-1,2-benzanthracene than in other groups, the incidence of tumours in the control series treated with Carbowax alone was so high that the possible carcinogenic activity of the spermicidal compounds could not be assessed.

We should like to thank Mr. R. O. Rees for help with these experiments. The research was supported by grants to the Chester Beatty Research Institute (Institute of Cancer Research : Royal Cancer Hospital) from the Medical Research Council, the British Empire Cancer Campaign, the Jane Coffin Childs Memorial Fund for Medical Research, the Anna Fuller Fund, and the National Cancer Institute of the National Institutes of Health, U.S. Public Health Service.

### REFERENCES

ALLEN, M. J., BOYLAND, E., DUKES, C. E., HORNING, E. S. AND WATSON, J. G.—(1957) *Brit. J. Cancer*, **11**, 212.
BACHMAN, W. E., KENNAWAY, E. C. AND KENNAWAY, N. M.—(1938) *Yale J. Biol. Med.*, **11**, 97.
GARDNER, W. V.—(1959) *Cancer Res.*, **19**, 170.
GLUCKSMANN, A. AND CHERRY, C. P.—(1958) *Brit. J. Cancer*, **12**, 32.
HOCH-LIGETI, C.—(1957) *J. nat. Cancer Inst.*, **18**, 661.
HUEPER, W. C.—(1956) *Proc. Amer. Ass. Cancer Res.*, **2**, 120.
LEHMAN, A., LUSKY, M. C. AND NELSON, A. A.—(1957) *Fed. Proc.*, **16**, 318.
PRATT-THOMAS, H. R., HEINS, H. C., LATHAM, E., DENNIS, E. J. AND McIVER, F.—(1956) *Cancer*, **9**, 671.
SCARPELLI, P. G. AND HAAM, EVON.—(1960) *Progr. exp. Tumor Res.* (Basle, Karger.) **1**, 179.
TEUTSCHLÄNDER, K.—(1926) *Z. Krebsforsch.*, **23**, 161.